# Is high flow nasal therapy still warranted for patients with AECOPD and acute hypercapnic respiratory failure?

**DOI:** 10.1186/s13613-025-01511-6

**Published:** 2025-07-07

**Authors:** Jinlv Qin, Guizuo Wang, Yixing Liao, Wenli Shang, Dong Han

**Affiliations:** 1 Radioimmunoassay Center, Shaanxi Provincial People’s Hospital, No. 256, West Youyi Road, Xi’an, Shaanxi 710068 China; 2 Department of Respiratory and Critical Care Medicine, Shaanxi Provincial People’s Hospital, No. 256, West Youyi Road, Xi’an, Shaanxi 710068 China; 3 Department of Critical Care Medicine, The First Affiliated Hospital, Zhejiang University School of Medicine, Hangzhou, Zhejiang 310003 China

To the Editor:

We thank Castro-Sayat et al. for their interest in our study [1]. In their comments [2] Castro-Sayat et al. emphasize that the management of respiratory failure in acute exacerbation of chronic obstructive pulmonary disease (AECOPD) is inherently complex and requires an individualized approach that goes beyond binary treatment comparisons. They correctly point out the difficulties of managing hypercapnic acute respiratory failure (ARF) in AECOPD, and we fully agree that our findings cannot be interpreted as a call against using high-flow nasal cannula (HFNC) in patients with chronic obstructive pulmonary disease (COPD) and hypercapnic ARF.

First, we agree that noninvasive ventilation (NIV) (alternating with HFNC) represents a clinical trend, which we also mentioned in discussion [1]. In mechanically ventilated patients at high risk of extubation failure, the use of HFNC with NIV after extubation significantly reduced the risk of reintubation compared with HFNC alone [3]. But in critically ill immunocompromised patients with ARF, the intubation and mortality rate did not differ between HFNC alone and NIV alternating with HFNC [4]. Therefore, the application of NIV (alternating with HFNC) in acute respiratory failure, particularly in hypercapnic ARF in AECOPD, still requires validation by large-scale randomized controlled trials (RCTs). At present, it may not be concluded that the combination of NIV and HFNC is necessarily superior to NIV or HFNC alone. Moreover, the optimal protocol for NIV combined with HFNC remains unclear (which modality to use first, specific duration allocation, etc.).

Second, diaphragmatic function was not the focus of this meta-analysis, and we provided baseline PaCO_2_ levels in Table 1. Patients who respond well to HFNC may have shorter ICU and hospital stays, as well as reduced hospital costs [5]but there are currently no definitive indicators to predict a patient’s response to HFNC. In the propensity-matched study mentioned by Castro-Sayat et al. (44 patients from the HFNC group and 44 from the NIV group) [5]the treatment failure rate was much higher in the HFNC group than in the NIV group (38.6% versus 11.4%, *p* = 0.003). Given the small sample size and non-RCT study design, it may be premature to conclude that HFNC failure does not affect prognosis.

Third, our meta-analysis included a small number of studies, a limitation that may reduce statistical power and affect the detection of meaningful clinical differences between HFNC and NIV. The use of NIV and HFNC in patients with hypercapnic ARF requires further investigation.

Fourth, we agree with Castro-Sayat et al. that in terms of respiratory support strategies for AECOPD patients, more flexible and individualized treatment options may be needed in the future, along with more specific criteria and methods to determine the most suitable treatment for each patient (HFNC, NIV, NIV + HFNC, etc.).

Finally, we would like to thank Castro-Sayat et al. again for their letter, which has provided us with the opportunity to offer additional perspectives to enhance the interpretation of our findings. Our findings should not be simply interpreted as opposing the use of HFNC in patients with COPD complicated by hypercapnic ARF. Instead, in this specific population, the selection and combination of HFNC and NIV require more specific criteria and methods.

## Data Availability

Not applicable.
